# Spontaneous Remission of Epileptic Seizures Following Norovirus Infection in a Patient With DNM1 Encephalopathy

**DOI:** 10.7759/cureus.60748

**Published:** 2024-05-21

**Authors:** Kazuo Kubota, Miho Adachi, Hidehiko Fujii, Hirotomo Saitsu, Hidenori Ohnishi

**Affiliations:** 1 Department of Pediatrics, Graduate School of Medicine, Gifu University, Gifu, JPN; 2 Department of Pediatrics, Ogaki Municipal Hospital, Ogaki, JPN; 3 Department of Biochemistry, Hamamatsu University School of Medicine, Hamamatsu, JPN

**Keywords:** whole-exome dna sequencing, norovirus, epileptic seizures, encephalopathy, dynamin 1

## Abstract

Epileptic seizures can be worsened by infections; however, they sometimes disappear or decrease after an acute viral infection, although this is rare. We report the spontaneous remission of epileptic seizures following norovirus-induced viral gastroenteritis in a boy with *DNM1* encephalopathy. He had clonic seizures daily from the age of two months and developed epileptic spasms at 14 months of age; he was admitted to the hospital at this time. A physical examination revealed hypotonia, strabismus, tongue protrusion with drooping, and widely spaced teeth. Although brain magnetic resonance imaging was unremarkable, electroencephalography revealed frequent occipital spikes. Three days after admission, the patient developed frequent diarrhea without a fever. A rapid immunochromatographic test of norovirus in a stool sample was positive. Immediately after the appearance of diarrhea, the epileptic seizures disappeared. Currently, at the age of five years, the patient has a profound psychomotor developmental delay; he has no verbal expression and is unable to walk. He has experienced involuntary movements of the myoclonus since 10 months of age. Whole-exome sequencing of the patient’s DNA revealed the presence of a heterozygous de novo variant of *DNM1*: c.709C>T (p.Arg237Trp). Although the findings from our patient suggest that underlying neural network abnormalities were ameliorated by immunological mechanisms as a result of the viral infection, further research is needed to clarify the mechanisms behind this spontaneous remission of seizures.

## Introduction

In children with epilepsy, viral infections can often worsen seizures. One of these viral infections is norovirus. Norovirus infection is the common cause of gastroenteritis, leading to vomiting and diarrhea. However, it has also been reported that epileptic seizures, especially those associated with infantile epileptic spasms syndrome, can disappear or decrease after viral infections, albeit rarely [1,2].

The *DNM1 *gene encodes dynamin 1, a GTPase that plays a crucial role in the catalysis of clathrin-mediated endocytosis and synaptic vesicle recycling, which is necessary for signaling pathway function and central nervous system development. Heterozygous variants in *DNM1 *are associated with epileptic encephalopathy, such as infantile epileptic spasms syndrome and Lennox-Gastaut syndrome. Patients with *DNM1 *variants reportedly have severe intellectual disability, a lack of speech, hypotonia, and an inability to walk [3].

We herein report the spontaneous remission of epileptic seizures following norovirus-induced viral gastroenteritis in a patient carrying a *DNM1 *variant.

## Case presentation

A boy was the first child of healthy, nonconsanguineous parents. He was born at 41 gestational weeks by normal vaginal delivery following an uneventful pregnancy. His father’s sister had Turner syndrome. He experienced daily clonic seizures from two months of age; these were treated with carbamazepine and phenobarbital. Additionally, he had myoclonus at 10 months of age. His psychomotor development was delayed; he acquired head control at four months, rolled over at seven months, and was able to sit with support at 13 months. The patient was admitted to the hospital at 14 months because he developed frequent seizures that were suspected epileptic spasms, consisting of brief tonic contractions of the axial muscles. These occurred two to three times daily, with each series consisting of 10-30 seizures. After their onset, motor regression occurred, and the patient was unable to roll over or sit with support.

**Figure 1:**
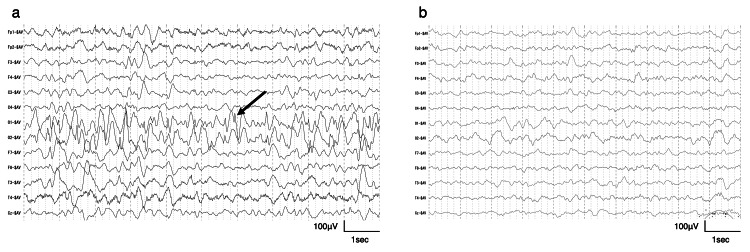
Drug-induced sleep electroencephalogram findings (a) Electroencephalogram showing frequent occipital spikes (black arrow) at 14 months of age. (b) No spikes were evident at three years and four months of age.

Physical examination revealed hypotonia, strabismus, tongue protrusion with drooping, and widely spaced teeth. Brain magnetic resonance imaging findings were unremarkable. An electroencephalogram revealed frequent occipital spikes without hypsarrhythmia (Figure 1a). Three days after admission for the treatment of epilepsy, the patient developed frequent diarrhea with no fever. A rapid immunochromatographic test of a stool sample was positive for norovirus. The diarrhea improved over approximately one week. Immediately thereafter, the patient’s seizures disappeared spontaneously without changes to antiepileptic drugs. To date, he has experienced no seizures for approximately four years, and an electroencephalogram at the age of three years and four months revealed no spikes (Figure 1b). However, myoclonus remained present and was treated with clonazepam and valproic acid. The patient regained the ability to roll over at one year and 11 months and crawled at four years. Currently, at the age of five years, he has a profound psychomotor developmental delay; he has no verbal expression and is unable to walk. Whole-exome DNA sequencing revealed that he is heterozygous for a known pathogenic* DNM1 *variant (NM_004408.4: c.709C>T, p.Arg237Trp). His parents lack the variant, indicating that it occurred de novo.

## Discussion

In children with epilepsy, acute viral infection often worsens epileptic seizures. Moreover, both norovirus- and rotavirus-induced gastroenteritis are known inducers of seizures in children [4,5]. However, some viral infections, including exanthema subitum, rotavirus colitis, and measles or herpes stomatitis, may also improve intractable seizures [6]. For example, Hattori reported that 86% of spontaneous seizure remissions are preceded by viral infections such as exanthema subitum, rotavirus gastroenteritis, measles, or chickenpox [1]. Similarly, Yamamoto et al. reported the disappearance of epileptic seizures subsequent to viral infections such as exanthema subitum, rotavirus colitis, measles, or mumps [2]. In our patient with *DNM1* encephalopathy, we observed the spontaneous remission of epileptic seizures following norovirus infection.

The mechanisms by which viral infection may affect the pathophysiology of epilepsy are unknown. Moreover, the mechanism by which epileptic seizures are kept in remission for a long time is also unclear. Individuals with norovirus infection exhibit early elevation of chemokines such as IL-8 and monocyte chemoattractant protein-1 and a persistent elevation of IL-10 [7]. IL-10 expression protects neurons and glia in the brain, mainly by inhibiting proapoptotic cytokines and stimulating protective signaling reactions [8]. Although IL-10 might be related to the cessation of epileptic spasms, we did not perform immunological investigations in the present case. However, we speculate that underlying neural network abnormalities were ameliorated by immunological mechanisms caused by the viral infection rather than by a specific immune response to *DNM1 *encephalopathy.

Pathogenic *DNM1 *variants affect brain development and function and cause epileptic encephalopathy with severe neurodevelopmental complications [3,9]. A c.709C>T variant in *DNM1 *was identified in our patient; this has previously been reported as a common variant. For example, Li et al. reported that c.709C>T was the most common variant in patients with *DNM1 *variants (identified in eight of 33 patients) [10], and von Spiczak et al. reported that it was the most common pathogenic variant in patients with *DNM1 *encephalopathy. Patients carrying this variant have homogeneous phenotypes, displaying infantile spasms with developmental delay before seizure onset and progressing to refractory epilepsy and movement disorders such as hyperkinetic movement and dystonia [11]. Similarly, our patient experienced epileptic seizures with suspected epileptic spasms, severe developmental delay, and myoclonus.

Epilepsy is generally intractable in patients carrying *DNM1 *variants, and the efficacy of antiepileptic drugs is limited. Notably, although many genetic variants have been identified in patients with epileptic encephalopathy using next-generation sequencing [12], there have been no reports of the genetic basis of patients who experience spontaneous remission of intractable seizures following infection. The clarification of this point may lead to new therapeutic options for epileptic seizures.

## Conclusions

We have reported a patient with epileptic seizures caused by *DNM1* encephalopathy that spontaneously disappeared following a norovirus infection. Epileptic seizures can disappear after an acute viral infection. Although our findings suggest that underlying neural network abnormalities may have been ameliorated by immunological mechanisms caused by the viral infection, further research is needed to clarify the mechanisms behind this spontaneous remission of seizures.
